# Role of locoregional surgery in patients with de novo stage IV breast cancer: analysis of real-world data from China

**DOI:** 10.1038/s41598-020-75119-0

**Published:** 2020-10-22

**Authors:** Li Ma, Yunzhe Mi, Shude Cui, Haibo Wang, Peifen Fu, Yongmei Yin, Feng Jin, Jianbin Li, Yinhua Liu, Zhimin Fan, Haiqing Zhang, Cuizhi Geng, Zefei Jiang

**Affiliations:** 1 Breast Center Department, The Fourth Hospital of Hebei Medical University, Shijiazhuang, China; 2 Breast Center Department, Henan Cancer Hospital, Zhengzhou, China; 3 Breast Center Department, Affiliated Hospital of Qingdao University, Qingdao, China; 4 Breast Center Department, The First Affiliated Hospital of Zhejiang University, Hangzhou, China; 5 Department of Oncology, Jiangsu Province Hospital, Nanjing, China; 6 Department of Breast Surgery, The First Affiliated Hospital of China Medical University, Shenyang, China; 7 Department of Breast Cancer, The Fifth Medical Center of the General Hospital of the Chinese People’s Liberation Army, Beijing, China; 8 Department of General Surgery, The First Hospital of Peking University, Beijing, China; 9 Department of Breast Surgery, The First Hospital of Jilin University, Jilin, China; 10 Department of Breast and Thyroid, Dalian Central Hospital, Dalian, China

**Keywords:** Breast cancer, Tumour immunology

## Abstract

Stage IV breast cancer is metastatic breast cancer (MBC). Because real-world data are lacking in China, our research attempts to explore the effect of locoregional surgery on the prognosis of patients with MBC. A total of 987 patients from 10 hospitals and 2 databases in East China (2004–2018) were included in this study. Overall, 47% of patients underwent locoregional surgery, and 53% did not. Surgeons tended to perform surgery on patients with small tumours (T1/T2), positive hormone receptor (HR) markers, and metastatic sites confined to a single organ and non-visceral sites (bone only/others) (each p < 0.05). Kaplan–Meier survival curves and the log-rank test showed that median survival was longer for patients who had locoregional surgery than for those who did not (45.00 vs. 28.00 months; *p* < 0.001). Patients who underwent surgery after systemic treatment had better survival than those who underwent surgery immediately (*p* < 0.001). In most subgroups, overall survival (OS) was significantly longer in the surgery group than in the no-surgery group (each *p* < 0.05), except for brain metastases and triple negative breast cancer. Therefore, we concluded that locoregional surgery for the primary tumour in MBC patients was associated with a marked reduction in risk of dying except for patients with brain metastases or triple-negative subtype.

## Introduction

Stage IV breast cancer is metastatic breast cancer (MBC) and a type of advanced breast cancer (ABC). MBC occurs when breast cancer cells spread beyond the breast, chest wall, and local lymph nodes^1^. The National Cancer Institute's SEER database report showed that from 1988 to 2003, de novo stage IV breast cancer accounted for approximately 3.5% of breast cancer cases^2^. A multicentre study showed that 2.4% of new breast cancer patients in China were MBC. The proportion in low-income areas were higher^3^. MBC is still an almost incurable disease with a median overall survival (OS) of 3 years, and the 5-year survival rate is only 25%^4^. The MBC Decade Report^4^ showed that progress has been slow in improving prognosis, quality of life, awareness and information about MBC. Recently, OS has improved, mostly due to the progress of treatment of human epidermal growth factor receptor 2 (HER2)-positive MBC^5^.

The goal of MBC treatment is to prolong survival, control tumour burden, reduce tumour-related symptoms and complications, and improve quality of life^1^. However, whether the primary tumour in MBC patients can benefit from locoregional surgery has been the focus of academic debate. A meta-analysis based on clinical retrospective studies and a retrospective analysis of the SEER database suggested that patients with MBC can benefit from local surgical treatment^2,6^. However, at the 2016 American Society of Clinical Oncology (ASCO) meeting, three phase III prospective clinical studies published their results. The MF07-01 study in Turkey revealed a positive result, especially for patients with simple bone metastases, while the study from Tata in India and the TBCRC 013 study in the US suggest that local surgery is not beneficial. The recent high-profile trial ECOG-ACRIN 2108 (E2018) also showed that early local therapy does not improve survival in patients with de novo stage IV breast cancer^7–10^.

No real-world study (RWS) has been conducted in China thus far. This study intends to extract the data of patients with de novo stage IV breast cancer from a multicentre database. Through the retrospective study of a large dataset of samples from the real world, we can explore the current status and specific characteristics of treatment for these patients in China. In particular, we aimed to determine the impact of locoregional surgery on the prognosis of patients with de novo stage IV breast cancer, with the goal of providing more references for standardized treatment of breast cancer patients.

## Patients and methods

We performed a population-based retrospective cohort study of patients with primary stage IV breast cancer. These patients were recorded in the China Society of Clinical Oncology breast cancer (CSCO BC) database and the Hebei Breast Cancer Center database with a diagnosis date between January 1, 2004, and December 31, 2018. In the CSCO BC database, patients with the primary tumour in MBC comprised approximately 6% of the database; 10 hospitals in 7 cities were included. These 7 cities (Beijing, Shijiazhuang, Zhengzhou, Qingdao, Hangzhou, Jilin, Dalian) are all located in East China.

We compared patients with de novo stage IV breast cancer who underwent locoregional surgery for their breast tumour to patients who did not. We excluded all patients with second tumour or unknown follow-up information and those whose metastatic site was unclear. Patients who received any type of surgical resection of the primary breast tumour (mastectomy or partial mastectomy) were categorized as having had surgery. Patients who did not receive any formal resection of their primary tumour or who underwent only breast biopsies for tissue diagnosis were categorized as not having surgery. If surgery status was not recorded, the participant was excluded.

Demographic information included age and year of diagnosis. Age was grouped into < 55 years and ≥ 55 years. Tumour characteristics used for classification were size; hormone receptor (HR), including oestrogen receptor (ER) and progesterone receptor (PR); human epidermal growth factor receptor 2 (HER2); and metastatic site. For patients with multiple organ metastases, each patient was counted only once, and when there was brain metastasis, only brain metastasis was counted. Tumour grade, radiotherapy, margin status after primary tumour resection and the number of metastatic lesions in transfer organs were poorly recorded in the CSCO BC database and Hebei Breast Center Database. Survival time was calculated in the database set using the date of diagnosis and one of the following: date of death, date last known to be alive, or date used as a cut-off for this file (April 1, 2019). The above grouping method and description refer in part to a study from the SEER database^2^.

### Statistical analysis

The χ^2^ test was used to compare the distribution of patient demographic characteristics and tumor related characteristics between the surgery and no surgery groups. Kaplan–Meier survival curves were generated to compare differences in survival probabilities over time between the surgery and no surgery groups, and the log-rank test was used to determine whether the survival curves were different between the two groups. Cox regression models were generated to describe the relationship between surgery and risk of death among patients with stage IV breast cancer; these models were used to calculate the hazard ratio (HR) and 95% confidence interval (95% CI) *p* values < 0.05 were considered to be statistically significant. All analyses were conducted by SpSS 21 statistical software, all figures were made by Graphpad prism 5. The above Statistical Analysis refer in part to a study of the SEER database^2^.

### Ethical approval

This study was approved by ethics committee of the Fourth Hospital of Hebei Medical University and all methods were carried out in accordance with the ethical rules of the Helsinki Declaration and Good Clinical Practice. Written informed consent was waived by the ethics committee of the Fourth Hospital of Hebei Medical University because this was a retrospective, non-invasive and observational study.

## Results

After eliminating disqualified data, there were 987 patients with de novo stage IV breast cancer between 2004 and 2018. A total of 506 cases from the CSCO BC database and 481 cases from the Hebei Breast Center Database were included. A total of 463 (47%) had surgery on their breast tumour, and 524 (53%) did not. Among patients who had surgery, 74 (16%) had partial mastectomies, and 389 (84%) had mastectomies. The demographics and tumour characteristics of patients in the surgery (S) and no-surgery (NS) groups are compared in Table 1.

**Table 1:** Characteristics of women diagnosed with first primary stage IV breast cancer included in the 2004–2018 CSCO BC database and Hebei breast center database.

Characteristic	Surgery, n (%) (n = 463)	No surgery, n (%) (n = 524)	*p* value
**Age (year)**			0.26
Median	55	56	
Range	24–73	24–93	
< 55	227 (49.0)	238 (45.4)	
≥ 55	236 (51.0)	286 (54.6)	
**Year of diagnosis**			< 0.01
2004–2008	58 (12.5)	23 (4.4)	
2009–2013	150 (32.4)	164 (31.3)	
2014–2018	255 (55.1)	337 (64.3)	
**Tumor size**			< 0.01
T1/T2	250 (54.0)	200 (38.2)	
T3/T4	127 (27.4)	250 (47.7)	
Unknown	86 (18.6)	74 (14.1)	
**Hormone receptor (HR)**			0.01
HR+	300 (64.8)	317 (60.5)	
HR−	123 (26.6)	130 (24.8)	
Unknown	40 (8.6)	77 (14.7)	
**Human epidermal growth factor receptor-2 (HER-2)**			0.85
HER2+	123 (26.6)	141 (26.9)	
HER2−	278 (60.0)	307 (58.6)	
Unknown	62 (13.4)	76 (14.5)	
**Subtype**			0.72
HR+	210 (45.4)	243 (46.4)	
HER2+	123 (26.6)	141 (26.9)	
Triple negative (TN)	65 (14.0)	61 (11.6)	
Unknown	65 (14.0)	79 (15.1)	
**Metastatic number**			< 0.01
1 Organ	326 (74.7)	302 (60.7)	
> 1 Organ	137 (25.3)	222 (29.3)	
**Metastatic site**			
Bone	179 (38.7)	171 (32.6)	< 0.01
Pulmonary/liver	218 (47.1)	302 (57.6)	
Brain	10 (2.2)	26 (5.0)	
Others	56 (12.1)	25 (4.8)	

The median age at diagnosis in the S group was 55 years (range 24–73 years) compared with 56 years (range 24–93 years) in the NS group. There was no significant difference between the two groups (*p* = 0.26). The numbers of patients who underwent surgery from 2014–2018, 2009–2013 and 2004–2008 were significantly different (*p* < 0.01). We observed a downward trend in the number of operations, indicating that surgeons were more cautious in choosing surgery over time. Among the patients who underwent surgery, their tumours were more likely to be ≤ 5 cm (T1/T2) (*p* = 0.01) and to have positive HR markers (*p* < 0.01) than the tumours of patients who did not undergo surgery. Additionally, surgeons tended to choose patients with metastatic sites confined to a single organ and non-visceral sites (bone only/others) (each *p* < 0.01). Immunohistochemical types and tumour markers (Her2) were not significantly different between the two groups.

**Figure 1:**
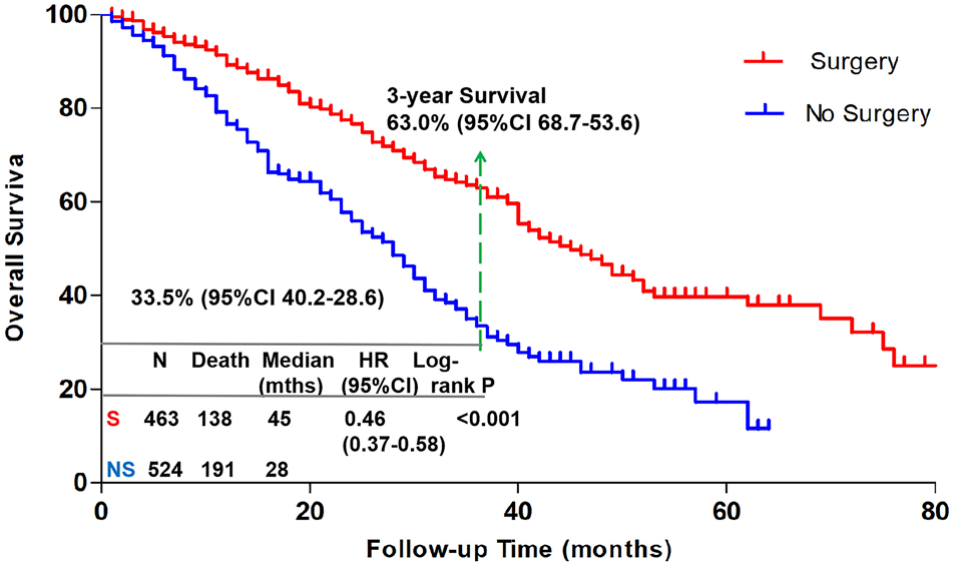
Overall survival by surgery status. Kaplan–Meier curves show overall survival in the surgery (red) and no surgery (blue) groups.

**Figure 2:**
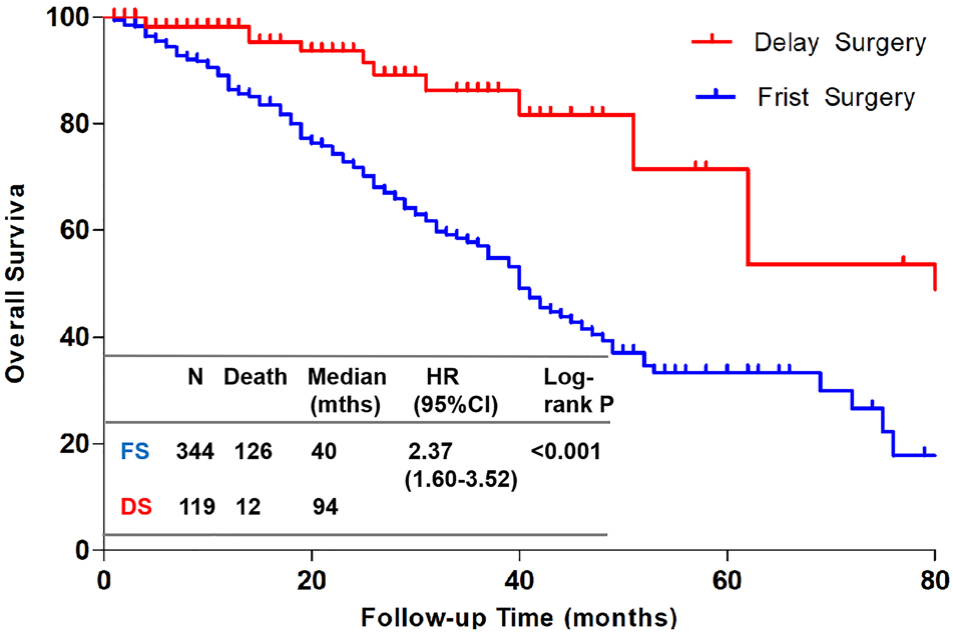
Overall survival by surgery status. Kaplan–Meier curves show overall survival in the delay surgery (red) and frist surgery (blue) groups.

Figure 1 shows a cumulative survival curve comparing the survival probability between the S and NS groups. These curves show that patients with MBC who underwent surgery had better survival rates than patients who did not. The log-rank test showed that the two curves were significantly different (*p* < 0.001). Median survival was significantly longer for patients who underwent surgery than for patients who did not (45.00 vs. 28.00 months; *p* < 0.001). The 3-year survival was 63.0% (95% CI 68.7–53.6%) in the S group vs. 33.5% (95% CI 42.0–28.6%) in the NS group. In the S group, 344 patients (74%) had local surgery immediately, referred to as the first surgery (FS) group, and 119 patients (26%) had local surgery after systemic treatment, referred to as the delayed surgery (DS) group. Figure 2 shows the cumulative survival curve comparing the survival probability of the FS group to that of the DS group. These curves indicate that patients who underwent surgery after systemic treatment had better survival than those who underwent surgery immediately. The log-rank test showed that the two curves were significantly different (*p* < 0.001). Median survival was significantly longer for DS than for FS (94.00 vs. 40.00 months; *p* < 0.001).

Univariate analyses showed that age ≥ 55 years, 2004–2008 diagnosis, larger tumour size (T3/T4), and concomitant brain metastasis or pulmonary/liver metastasis were associated with an increased risk of death (Table 2). In a multivariate Cox proportional model with significant baseline characteristics, survival was independent of greater tumour size (T3-T4) (HR 2.33; 95% CI 1.54–3.53; *p* < 0.001) and bone-only metastasis at initial presentation (0.70; 95% CI 0.56–0.88; *p* < 0.001). However, age < 55 years (HR 0.84; 95% CI 0.68–1.04; p = 0.113) was not associated with OS (Table 3). The crude hazard ratio for the association between surgery and death during follow-up was 0.45 (95% CI 0.36–0.55), and the adjusted hazard ratio was 0.51 (95% CI 0.41–0.64), indicating that patients in the S group were almost 49% less likely to die during the study period than patients in the NS group.

**Table 2:** Unadjusted associations among women diagnosed with first primary stage IV breast cancer, 2004–2018.

Characteristic	n	cHR (95% CI)
**Age (year)**		
< 55	465	1.00
≥ 55	522	1.16 (1.16–1.65)
**Year of diagnosis**		
2004–2008	81	1.00
2009–2013	314	0.52 (0.34–0.80)
2014–2018	592	0.72 (0.58–0.91)
**Tumor size**		
T1/T2	450	1.00
T3/T4	337	2.34 (1.55–3.54)
Unknown	160	3.63 (2.41–5.46)
**Hormone receptor (HR)**		
HR+	617	1.00
HR−	253	0.70 (0.51–0.94)
Unknown	117	0.71 (0.50–1.02)
**Human epidermal growth factor receptor-2 (HER-2)**		
HER2+	264	1.00
HER2−	585	0.88 (0.64–1.23)
Unknown	138	0.78 (0.58–1.04)
**Subtype**		
HR+	453	1.00
HER2+	264	0.85 (0.63–1.16)
Triple negative (TN)	126	0.95 (0.68–1.32)
Unknown	144	0.84 (0.55–1.27)
**Metastatic number**		
1 Organ	628	1.00
> 1 Organ	359	0.85 (0.67–1.08)
**Metastatic site**		
Bone only	350	1.00
Pulmonary/liver	520	1.71 (1.06–2.75)
Brain	36	3.15 (1.98–5.01)
Others	81	5.65 (3.05–10.46)
**Surgery**		
No surgery	524	1.00
Surgery	463	0.45 (0.36–0.55)

**Table 3:** Uni- and multivariate Cox model analysis of clinically important parameters for overall survival.

Parameter	HR	95% CI	*p* value	HR_adj_^a^	95% CI	*p* value
Age < 55 years	0.74	0.60–0.92	0.006	0.84	0.68–1.04	0.113
T3/T4	2.34	1.55–3.54	< 0.001	2.33	1.54–3.53	< 0.001
Bone only	0.63	0.50–0.79	< 0.001	0.70	0.56–0.88	0.002
Surgery	0.45	0.36–0.55	< 0.001	0.51	0.41–0.64	< 0.001

^a^Adjusted HR results from multivariate Cox Models with all significant baseline included in the model.

**Figure 3:**
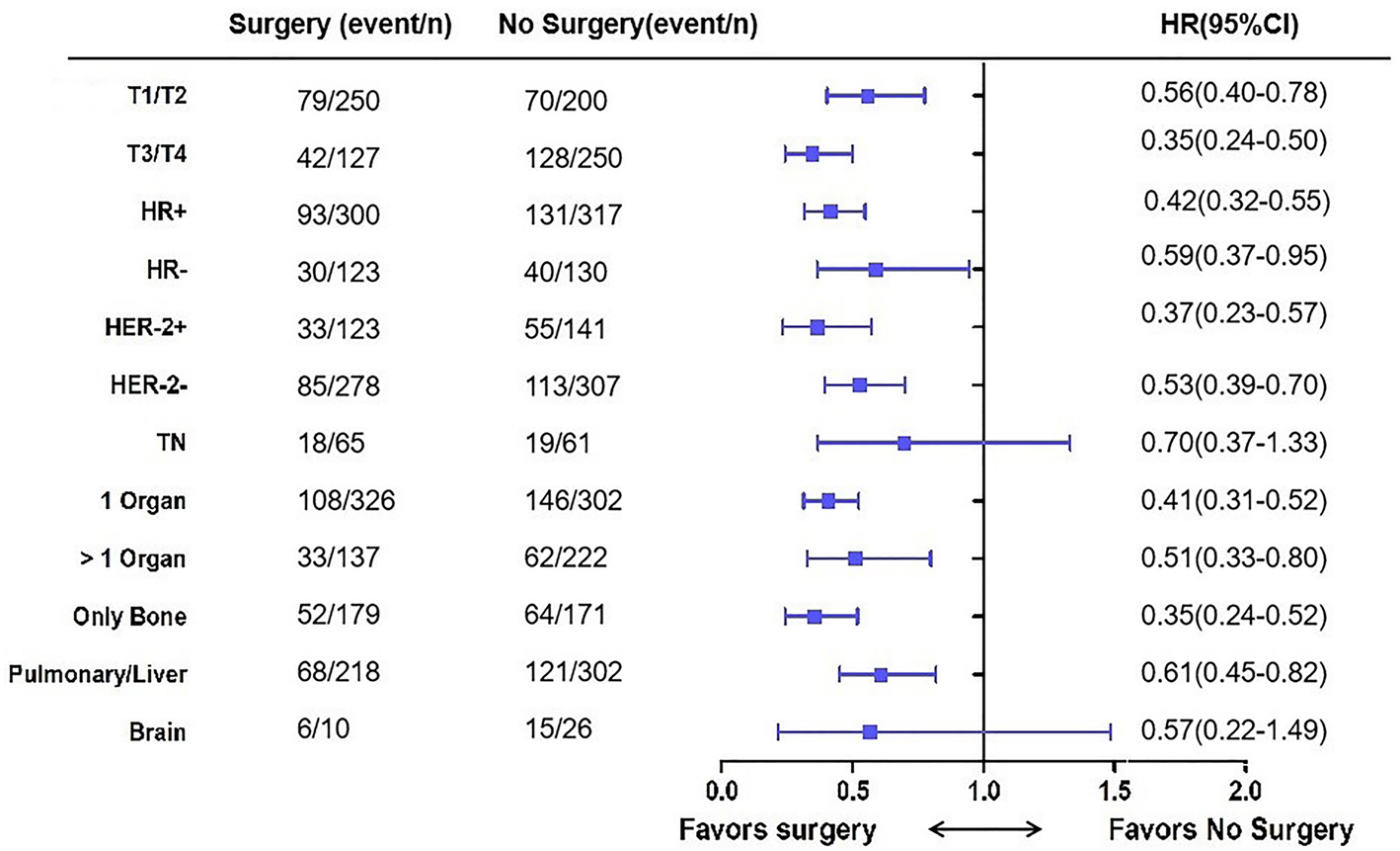
Forest plot of overall survival subgroup analyses with hazard ratios.

Subgroup analyses (Fig. 3) showed that in most subgroups, OS was significantly longer in the S group than in the NS group (each *p* < 0.05). However, patients with brain metastases and triple negative (TN) breast cancer did not benefit from surgery. In the bone-only metastasis subgroup, the 3-year survival rates were 73.4% (95% CI 66.0–82.9%) in the S group and 36.6% (95% CI 25.5–47.9%) in the NS group, with a 23-month longer median survival time (HR 0.32; 95% CI 0.21–0.48; *p* < 0.001).

## Discussion

To our knowledge, this is the first RWS in China to systematically analyse the treatment of patients with de novo stage IV breast cancer. Patients who were enrolled in the CSCO BC database and Hebei Breast Center Database between 2004 and 2018 had a better survival advantage after surgical resection of the primary breast tumour than patients who did not have surgery. Patients who underwent surgery were 54% less likely to die during the study period than patients who did not. Even among patients who died during follow-up, patients who underwent surgery on their primary breast tumour had a longer median survival than those who did not (21.5 vs. 14.0 months *p* < 0.001). The findings from the current study are similar to previously published retrospective studies^2,6,11–13^. Patients with MBC who underwent primary breast tumour resection have a survival advantage over patients who receive only systemic treatment.

Unlike retrospective studies, the results of prospective studies are controversial, and the only positive result was from the MF-07 clinical trial. A total of 271 patients with the primary tumour in MBC were enrolled, and the median follow-up period was 40 months. The risk of death was 34% lower in the local surgery group than in the NS group, with a 9-month longer median survival (46 months vs 37 months; *p* = 0.005). Unplanned subgroup analyses showed that the risk of death was significantly lower in the S group than in the NS group with respect to HR+ status, HER− status, patients younger than 55 years and patients with solitary bone metastases. It is worth noting that nearly 50% of patients had bone-only metastasis in the MF-07 study, but only 35% (69/196) of patients had been treated with bisphosphonates. The TBCRC 013 study found that there was no significant difference in median survival time between the S and NS groups (77 months vs 71 months). Similarly, the Indian TATA study followed up 350 patients with the primary tumour in MBC for 23 months and found that there was no significant difference in median OS between the S group and the NS group (19.2 months vs 20.5 months, respectively, *p* = 0.79). The 2-year survival rates were 41.9% and 43.0%, respectively. In terms of survival data, it is not difficult to see that the TATA study had the worst outcomes. This may be because there were more severe patients in the study (approximately 75% patients had more than 3 metastasis sites), and the incidence of the use of herceptin was too low (8%; 9/107). There was a great deviation between these studies and clinical practice.

E2108, a multicentre, phase 3 study, presented results at the plenary session of the ASCO 2020 virtual meeting. They found no statistical differences between the S and NS groups in OS (68.4% vs 67.9%, *p* = 0.63) and PFS (*p* = 0.40). The average OS for the entire study population was 54 months. However, locoregional recurrence or progression was 2.5 times higher in the NS group than in the S group (25.6% vs 10.2%; *p* = 0.003). Subgroup analyses showed that for 20 women with TNBC, survival was worse in the S group^10^. Although the results of the E2108 trial were different from ours, our results all showed that TNBC patients could not benefit from locoregional surgery. Similarly, no comparison was made between the patients with only bone metastases and other organs. We will continue to look forward to the results of the JCOG 1017 trial.

The TBCRC-013 and TATA studies both found that the risk of local progression-free survival (PFS) in the S group was lower but that the risk of distant PFS metastasis was higher. The results were similar to previous preclinical studies^14–17^, which showed that metastatic tumours grow after resection of the primary tumour. The mechanisms might include fresh surgical propagation, increased circulating tumour cell adhesion to vascular endothelium of the target organs, surgery-induced angiogenesis transition, surgery-induced immunosuppression and inflammation cascade^18,19^. However, in other aggressive metastatic cancers, such as ovarian and renal, associations between reduced tumour burden and improved survival have been observed^20–23^. Surgery can lower tumour burden and make chemotherapy more effective and can clear areas of necrotic tumours inaccessible to drugs^24^.

At present, for local surgery treatment of patients with MBC, common clinical guidelines still lack clear recommendations. In the absence of high-level evidence, the National Comprehensive Cancer Network (NCCN) breast cancer guidelines recommend surgery only to relieve local symptoms such as skin ulceration^25^, bleeding and pain. According to the European Society for Medical Oncology (ESMO) guidelines, patients with oligometastases who are sensitive to systematic treatment tend to be treated locally for the purpose of radical cure^26^.

We noticed that the MF-07 study was different from the TBCRC-013 study and the TATA study in that patients underwent surgery directly after admission. In our study, we also compared the timing of surgery. The results showed that the median survival time of patients undergoing surgery after systemic treatment was 54 months longer than that of patients undergoing surgery first. Although we do not have data on the sensitivity of these patients to systemic treatment, we still believe that locoregional surgery should be performed after systemic treatment. Regarding the better timing of surgical intervention, a retrospective study from the University of MD Anderson Cancer Center seems to give the answer, demonstrating that locoregional surgery of patients with MBC was associated with improved metastatic PFS when performed more than 3 months after diagnosis^27^. At the same time, the experimental design of E2108 also supports this view. In our study, we observed a downward trend in the rate of surgery from 2004 to 2018. This result is similar to the data released by Kelly Hunt at the 2019 ASCO meeting^28^. We think this is because surgeons have realized in recent years that if patients cannot benefit from systemic treatment, it may be more difficult for them to benefit from locoregional surgery.

The kind of patient that can benefit from the operation is the question that we have been exploring. In our study, subgroup analyses showed that in most subgroups, patients can benefit from surgery, except for patients with brain metastases and TN breast cancer. For immunohistochemical subtypes, HR+ and HER2− patients benefited from surgery in the MF-07 study. Regarding metastatic organs, patients with bone-only metastasis in the MF-07 study benefited more from local surgery. In our study, in patients with bone-only metastasis, surgery was able to extend the survival time by 23 months, and the 3-year survival rate was as high as 73.4%. Although patients with pulmonary or liver metastases still benefit from surgery in our study, the MF-07 study suggests that surgery can lead to worse outcomes in these patients.

Professors Hellman and Weichselbaum first proposed the concept of "oligometastases" in 1995^29^. In the ABC4 guidelines, oligometastasis is defined as low-volume metastatic disease with a limited number and size of metastatic lesions (up to five and not necessarily in the same organ), potentially amenable to local treatment aimed at achieving a complete remission status. Oligometastasis was considered a transfer of relative inertia and weak spreading ability, similar to the tumour having a milder character. Many previous studies have shown that patients with oligometastases of breast cancer can benefit from surgery or stereotactic radiotherapy (SRS)^30–32^.

Because the database lacks records of the resection margins and radiotherapy conditions, this study has not been able to analyse these results. The E2108 study found that the surgical margin remained positive in approximately 20%(22/109) of the locoregional surgery group. Moreover, surgery and radiation to the tumour do not extend OS compared with systemic treatment alone in women with stage IV breast cancer.

MBC treatment should not only pursue survival benefits but also consider quality of life and psychological needs. In clinical work, many patients hope to receive locoregional surgery because a palpable malignant tumour increases their inner fear. However, the E2108 study mentioned that the adverse effects of surgery and radiation appear to balance out the gains in quality of life that were achieved with better control of the primary tumour. The TBCRC 013 study mentioned that the performance of local surgery should be decided jointly between the doctor and the patients. Therefore, the full communication and individualized treatment of patients are very important^9^.

Randomized controlled trials (RCTs) are still the cornerstone of medical development. However, the applicability of the evidence generated from RCTs will depend on the degree of alignment between the research and the target population. The external generalizability of RWSs is stronger; RWSs represent a wider range of patients and have the potential to solve problems that cannot be solved in RCTs. Thus, our results provide important information to facilitate treatment decisions. However, there were still some important limitations in our research. Due to the observational design, it was inevitable that some missing data could not be retrieved. It is inevitable that there is selection bias and information bias in this study; only 10 hospitals representing the main provinces were included; thus, the results cannot reflect the overall situation in China.

Therefore, we concluded that locoregional surgery for the primary tumour in MBC patients was associated with a marked reduction in risk of dying expect for patients with brain metastases or triple-negative subtype.

## Data Availability

The datasets generated during and/or analyzed during the current study are available from the corresponding author on reasonable request.
